# Chemical composition and inhibitory effects of water extract of Henna leaves on reactive oxygen species, DNA scission and proliferation of cancer cells 

**DOI:** 10.17179/excli2016-429

**Published:** 2016-12-21

**Authors:** Manish Kumar, Madhu Chandel, Paramjeet Kaur, Kritika Pandit, Varinder Kaur, Sandeep Kaur, Satwinderjeet Kaur

**Affiliations:** 1 Department of Botanical and Environmental Sciences, Guru Nanak Dev University Amritsar-143005, Punjab, India; 2 Akal College of Basic Sciences (Botany), Eternal University, Baru Sahib, Sirmour-173101, Himachal Pradesh, India

**Keywords:** Lawsonia inermis L., antioxidant activity, cytotoxic, HPLC, polyphenols

## Abstract

From the centuries, *Lawsonia inermis* L. (Henna) is utilized in traditional health care system as a medicinal and cosmetic agent. The present study was intended to assess antiradical, DNA protective and antiproliferative activity of water extract of *Lawsonia inermis* L. leaves (W-LI). Antioxidant activity was estimated using various *in vitro* assays such as DPPH, ABTS, superoxide anion radical scavenging, FRAP, deoxyribose degradation and DNA protection assay. Growth inhibitory effects of W-LI were assessed using MTT assay against different cancer cell lines *viz.* HeLa, MCF-7, A549, C6 and COLO-205. From the results of antioxidant assays, it was found that W-LI quenched DPPH and ABTS cation radicals with IC_50_ value of 352.77 µg/ml and 380.87 µg/ml respectively. It demonstrated hydroxyl radical scavenging potential of 59.75 % at highest test dose of 1000 µg/ml in deoxyribose degradation assay. The results of FRAP assay showed that W-LI also possesses significant reducing activity. Extract inhibited hydroxyl radical induced pBR322 plasmid DNA strand scission, thus conferring DNA protection. Growth inhibition of various cancer cell lines was achieved to the varying extent on treatment with W-LI. Further, it was observed that activity was quite promising against colon cancer COLO-205 cells (GI_50_ 121.03 µg/ml). HPLC profiling of W-LI revealed the presence of different polyphenolic compounds such as ellagic acid, catechin, quercetin, kaempferol etc. which might be contributing towards antioxidant and cytotoxic activity. The present study demonstrated that polyphenols rich W-LI extract from leaves of *L. inermis* possesses ability to inhibit oxidative radicals and cancer cells proliferation.

## Introduction

A redox balance exists between ROS and antioxidants in normal cells. Any impairment in the balance results in the increased ROS production leading to the oxidative stress conditions in the cell. Normally, ROS play an important role in cell functioning but excessive generation of ROS can impose deleterious effects on the various biological molecules (Gulcin et al., 2005[33]; Fidan and Dundar, 2008[27]; Klaunig et al., 2010[48]). There are two main sources of oxidative stress viz. external and internal. The external sources include environmental pollutants, drugs, drinking of alcohol, tobacco smoking and radiations while the internal sources include ROS generation in organelles like mitochondria, peroxisomes etc. (del Rio et al., 1992[21]; Ebadi, 2001[23]; Inoue et al., 2003[40]; Kumar, 2011[51]; Gupta et al., 2012[34]). Mitochondrial respiratory chain is the main source of reactive oxygen species. Under hypoxic condition, mitochondrial respiratory chain generates nitric oxide leading to the production of various other reactive nitrogen species (RNS) (Poyton et al., 2009[67]). ROS possess ability to cause lesions in DNA leading to the alterations in its structure and coding properties (Ragu et al., 2007[68]; Degtyareva et al., 2008[20]). Interaction of ROS with biomolecules can also lead to the inhibition of normal DNA synthesis and repair due to inactivation of antioxidant proteins as well as enzymes (Eiberger et al., 2008[24]; Gillard et al., 2004[31]). Single strand and double strand breaks, oxidation of purines and pyrimidines and apurinic/apyrimidinic DNA sites are the typical outcomes of ROS incited oxidative DNA damage. 8-oxo-7,8-dihydroguanine (8-oxoGua) and 2,6-diamino-4-hydroxy-5-formamidopyrimidine are commonly known endogenous DNA modifications. These are produced due to addition of hydroxyl radical to the C8 position of guanine ring resulting in a 8-hydroxy-7,8-dihydroguanyl radical which on oxidation results in 8-oxoGua or on reduction can form ring-opened FapyGua (Spassky and Angelov, 1997[78]; Altieri et al., 2008[4]).

Every organism is endowed with internal antioxidant and repair systems that provide protection against damage. However, under high oxidative stress conditions, antioxidant defense system is unable to confer protection. Thus, antioxidants derived from natural plant products can be used to modulate the damaging effects of oxidative stress (Kanter, 1998[42]). Antioxidants are the substances which have capacity to quench various reactive species leading to the inhibition of initiation and propagation of chain reaction. Vast research has been carried out to identify natural antioxidants as they reduce oxidative damage (Malta et al., 2013[61]; Yuan et al., 2013[86]; Khole et al., 2014[45]). From the starting of 21^st^ century, natural products are regarded as source of prospective medicines (Cerella et al., 2014[13]; Gechev et al., 2014[29]; Georgiev, 2014[30]; Schnekenburger et al., 2014[72]). Phytoconstituents such as flavonoids, stilbenoids, alkaloids and polysaccharides have gained immense attention of scientific community across the world, due their numerous biological properties (Georgiev, 2014[30]; Lanzotti, 2014[54]; Xiao, 2015[84]). Plant polyphenolics are known to provide protection against oxidative damage and play great role in reducing the cancer risk (Hung et al., 2004[38]; Hung et al., 2015[37]). Various studies have demonstrated that plant extracts can hit multiple targets, thus rendering pleiotropic and synergistic effects (Lansky et al., 2005[53]; Machana et al., 2012[60]; Seeram et al., 2004[73]). Compounds *viz*. camptothecin, podophylotoxin and paclitaxel are some of the examples of plant derived drugs which have greatly influenced anticancer research (Khazir et al., 2014[44]). Many of the drugs derived from natural products are potent and exhibit little or no side effects (Ma and Wang, 2009[59]). Thus, it is need of time to explore more medicinal plants for various bioactive properties.

*Lawsonia inermis* L. is generally used for treatment of jaundice and renal lithiasis, against skin inflammation and measles, wounds healing etc. in traditional medicinal systems in various parts of the world (Chopra et al., 1956[16]; Bellakhdar, 1997[9]; Kumari et al., 2011[52]; Oladunmoye and Kehinde, 2011[65]; Sharma et al., 2012[74]). Earlier, we reported various other extract/fractions of *L. inermis* for antioxidant, anticancer and apoptosis inducing activity (Kumar et al., 2014[50], 2015[49]). The present study was undertaken to evaluate inhibitory effects of water extract from Henna leaves on reactive oxygen species, oxidative DNA damage and cancer cells growth.

## Materials and Methods

### Chemicals

Ethidium bromide, 2,2-Diphenyl-1-picrylhydrazyl (DPPH), Ferric chloride, Nicotinamide adenine dinucleotide (NADH), Phenazine methosulphate, Ascorbic Acid, 2-Thiobarbituric Acid and L-Ascorbic acid were obtained from HiMedia Pvt. Limited. Mumbai. 2-azinobis (3-ethylbenzothiazoline-6-sulfonate) (ABTS), TPTZ (2,4,6-tripyridyl-*s*-triazine), 3-(4,5-dimethylthiazol-2-yl)-2,5-diphenyltetrazolium bromide (MTT) and Deoxyribose were purchased from Sigma Chemical Co. (St Louis, MO, USA). pBR322 plasmid DNA was purchased from Genei Pvt. Ltd., Banglore (India). All other chemicals used in the present experimental study were of AR grade. 

### Collection of plant material and extraction

The plant material (leaves) of the *Lawsonia inermis* L. were obtained from the local market at Amritsar, Punjab, India. The plant was identified and submitted to the Herbarium of the Department of Botanical and Environmental Sciences, Guru Nanak Dev University, Amritsar, with accession no. 6773 (Kumar et al., 2014[50]). Leaves (1 kg) were finely powdered using grinder and percolated in double distilled water (3 L) for 2 days at room temperature. The miscella so obtained was concentrated using rotary vacuum evaporator (Buchi Rotavapor R-210) and lyophilized to obtain water extract which was named as W-LI.

## Phytochemical Analysis

### Total phenolic content (TPC assay)

The total phenolic content was calculated as gallic acid equivalents (GAE) in mg/g of dry sample by using the protocol of Yu et al. (2002[85]). 

### Total flavonoid content (TFC assay)

The total flavonoid content was then expressed as rutin equivalents (RE) in mg/g of dry sample by using the protocol of Kim et al. (2003[47]).

## High-Performance Liquid Chromatography (HPLC) Analysis

### Sample preparation

For high-performance liquid chromatography (HPLC) analysis, W-LI was prepared in methanol (HPLC grade) and filtered through a 0.22 μm Acrodisc® syringe filter (PALL, Life Sciences) and then injected into the UHPLC system.

### HPLC instrument and chromatographic conditions

HPLC analysis was performed on Shimadzu UHPLC Nexera system (Shimadzu, MA, USA), provided with a photodiode array (PDA) detector as described in Kumar et al. (2014[50]). Sample was analyzed using a gradient mobile phase consisting of 0.1 % acetic acid aqueous as solution A and methanol as solution B. The flow rate was set as 1 ml/min and the injection volume used was 5 μl. The sample detection was done at 280 nm. 

## Antioxidant Assays

### DPPH radical scavenging assay 

DPPH scavenging activity was determined using method of Blois (1958[12]) with minor modifications. Different concentrations (50-1000 μg/ml) of W-LI were added to 2 ml of 0.1 mM methanol solution of DPPH (2, 2-Diphenyl-1-picrylhydrazyl). After 30 minutes, absorbance of reaction mixture was taken at 517 nm using UV-VIS spectrophotometer (Systronics 2202 UV-Vis Spectrophotometer). 

Radical scavenging activity % = A_0 _- A_1_/A_0_ × 100 

where A_0_ is the absorbance of DPPH solution + vehicle solvent, A_1_ is the absorbance of reaction mixture. 

### ABTS^.+^ radical scavenging assay 

ABTS^.+^ scavenging assay was determined according to method of Re et al. (1999[70]) with minor modifications. The ABTS cation solution was diluted with ethanol, so that the absorbance of ethanolic solution become 0.70 (± 0.02) at 734 nm. Test solution (50-1000 μg/ml) was added to the diluted ethanolic ABTS cation solution and finally, absorbance was taken after 5 minutes (Systronics 2202 UV-Vis Spectrophotometer). 

Radical scavenging activity % = A_0_ - A_1_/A_0_ × 100 

where A_0_ is the absorbance of ethanolic ABTS radical cation solution, A_1_ is the absorbance of reaction mixture.

### Superoxide anion radical scavenging assay 

The superoxide anion radical scavenging activity of W-LI was determined by method described by Nishikimi et al. (1972[64]) with minor modifications. About 1 ml of nitroblue tetrazolium (NBT) solution, 1 ml of NADH solution and W-LI extract concentrations (50-1000 μg/ml) were mixed in test tubes. The reaction was started by adding 100 μl of phenazine methosulphate (PMS) solution (60 μM). The reaction mixture was incubated at 25 °C and the absorbance at 560 nm was measured against the control samples using UV-VIS spectrophotometer (Systronics 2202 UV-Vis Spectrophotometer, India). 

Antioxidant activity % = A_0_ - A_1_/A_0_ × 100 

where A_0_ is the absorbance of control, A_1_ is the absorbance of reaction mixture.

### Deoxyribose degradation assay

Deoxyribose degradation assay was carried out by the method of Halliwell et al. (1987[36]) and Arouma et al*. *(1987[7]) with slight modifications**.** In this assay, EDTA (1 mM), FeCl_3_ (10 mM), hydrogen peroxide (10 mM), 2-Deoxyribose (10 mM), a test sample (1 ml), phosphate buffer and Ascorbic acid (1 mM) were mixed in the test tubes. After that above obtained reaction mixture was incubated at 37 °C for 1 hour. After incubation time period, 1 ml of above mixture was mixed with 1 ml of TBA and TCA each. This is followed by heating reaction mixture at 80 °C on water bath for 90 minutes. Tubes were finally cooled and absorbance of the pink chromogen formed was measured spectrophotometrically at 532 nm using Elisa reader. 

Percent hydroxyl radical scavenging potential was calculated by formula as given below:

Radical scavenging activity (%) = A_0_ - A_1_/A_0_ × 100 

Where A_0_ is the absorbance of control, A_1_ is the absorbance of reaction mixture containing test sample.

### FRAP assay

The FRAP assay was performed using a method described by Benzie and Strain (1996[11]) with minor modifications. The working FRAP reagent was prepared by mixing 25 ml of 300 mM acetate buffer, pH 3.6, 2.5 ml of 10 mM tripyridyltriazine (TPTZ) in 40 mM hydrochloric acid and 2.5 ml of 20 mM ferric chloride. Different concentrations of W-LI extract (0.3 ml) were added to freshly FRAP reagent (3 ml). The absorption of the mixture was measured at 593 nm after 10 min. Increase in absorbance is indicative reducing ability of extract. 

### DNA protection assay

DNA protection assay was performed according of method of Lee et al. (2002[55]) with slight modifications. Supercoiled pBR322 plasmid DNA was incubated with Fenton's reagent (30 mM H_2_O_2_, 50 μM ascorbic acid and 80 μM FeCl_3_) in the presence/absence of extract concentrations. Final volume of the reaction mixture was raised up to 20 μl with autoclaved distilled water. Reaction mixtures were incubated for 30 min at 37 °C. After incubation, loading dye was added and contents of reaction mixture were loaded into the wells of agarose gel. Electrophoresis was carried out in TAE buffer. Plasmid DNA bands were analyzed by ethidium bromide staining using Gel Doc XR system (Bio-Rad, USA).

Percentage DNA in different bands was calculated on the basis of integrated density value after treatment of pBR322 plasmid DNA with Fenton's reagent and different concentrations of W-LI using AlphaEase FC software.

## Growth Inhibitory Studies

Various cancer cell lines *viz.* HeLa (Human cervical cancer), MCF-7 (Human breast adenocarcinoma), A549 (Human alveolar adenocarcinoma), COLO 205 (Human colon adenocarcinoma) and C6 glioma (Rat glioblastoma) cell lines were purchased from the National Centre for Cell Science (NCCS, Pune, India). Different cell lines were cultured in DMEM/RPMI medium containing 10 % FBS and antibiotic-antimycotic solution. Cells were grown at 37 ˚C and 5 % CO_2_ using CO_2_ incubator.

### MTT assay

MTT assay was carried out to assess cytotoxic potential of test samples using method of Mickisch et al. (1990[63]) with slight modifications. Cells were trypsinized and finally centrifuged to obtain cell pellet which is further suspended in fresh medium. Cells were counted using hemocytometer and seeded at density of 8000 cells per well of the 96 well plate. After time period of 24 h, cells were treated with test samples for next 24 h. On the completion of treatment time, MTT (5 mg/ml) was added and incubated for 2 h. Finally, media was removed from the wells and DMSO (100 µl) was added per well to dissolve the purple colored formazan crystals and finally reading was taken at 570 nm. 

The percentage growth inhibition was calculated using the formula:

% cytotoxicity = A_0_-A_1_/A_0_×100

Where A_0_ is the absorbance of control, A_1_ is the absorbance of test sample.

### Statistical analysis

The results were expressed as the average ± standard error. GI_50/_IC_50_ values were calculated using regression equation. The data was analyzed for statistical significance using analysis of variance (One-way ANOVA) and the difference among means was compared by highly significant difference (HSD) using Tukey's test. The significance of results was checked at *p ≤ 0.05.

## Results

### Phytochemical analysis

**Figure 1:**
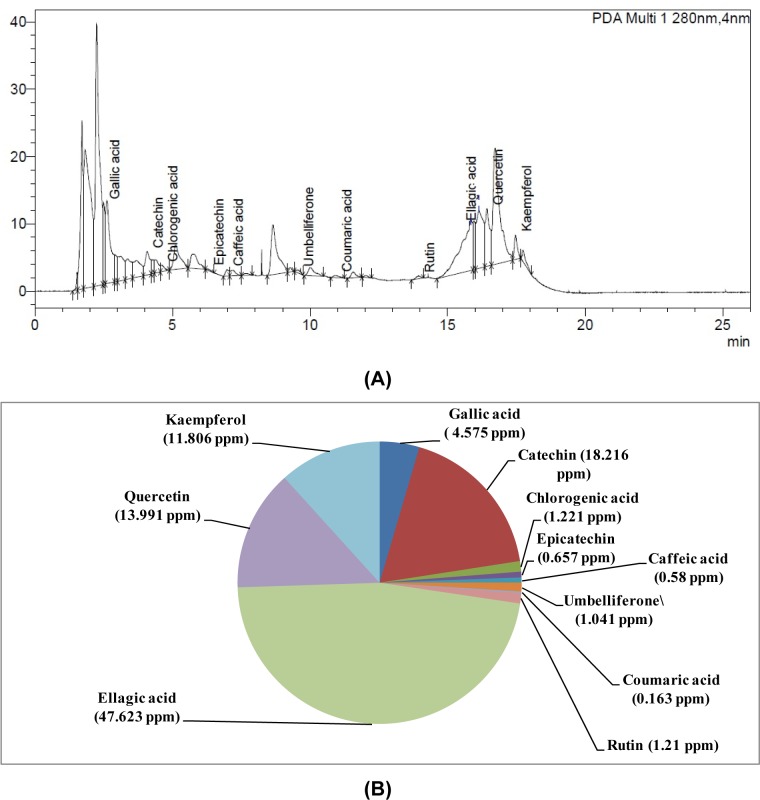
(A) HPLC chromatogram of W-LI extract from *Lawsonia inermis *leaves; (B) Quantitative analysis of phytochemicals using HPLC in W-LI extract from* Lawsonia inermis *leaves

W-LI showed phenolic content of 93.33 mg GAE/gram of dry weight of extract and flavonoid content of 68.16 mg RE/gram of dry weight of extract. Further, phytochemical identification and quantification using HPLC technique revealed the presence of gallic acid, catechin, chlorogenic acid, caffeic acid epicatechin, umbelliferone, coumaric acid, rutin, ellagic acid, quercetin and kaempferol in W-LI. Ellagic acid was found to be major constituent with concentration of 47.623 ppm followed by catechin (18.216 ppm), quercetin (13.991 ppm) and kaempferol (11.806 ppm). All other compounds were present in very low quantity (less than 10 ppm) (Figure 1(Fig. 1)).

### Antioxidant activity

**Figure 2:**
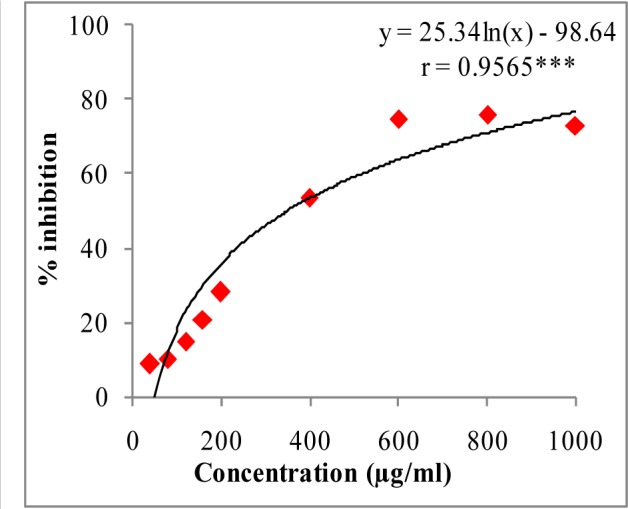
DPPH radical quenching activity of W-LI extract from *Lawsonia inermis *leaves. Regression analysis: ***represents significance at p≤ 0.001 One way ANOVA: F ratio = 435.95*; HSD = 6.52 (*p ≤ 0.05).

**Figure 3:**
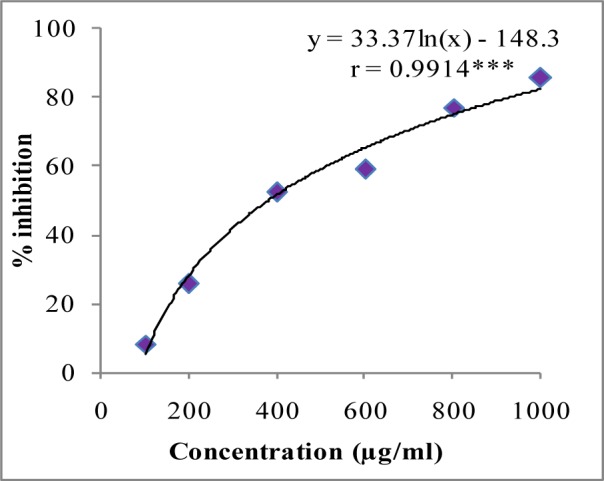
ABTS cation radical quenching activity of W-LI extract from *Lawsonia inermis *leaves. Regression analysis: ***represents significance at p≤ 0.001 One way ANOVA: F ratio = 611.83*; HSD = 5.69 (*p ≤ 0.05).

**Figure 4:**
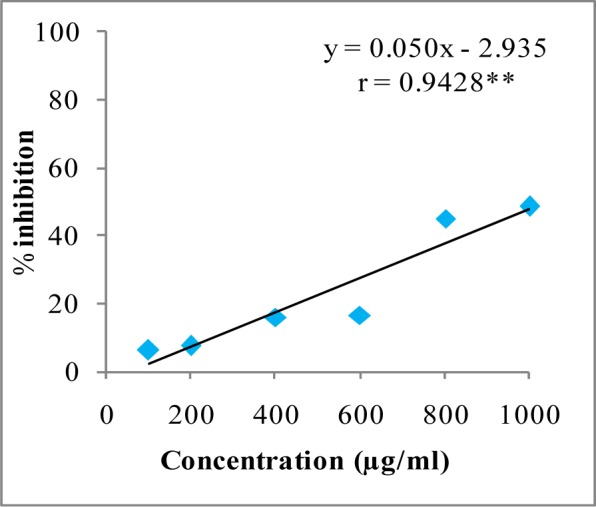
Superoxide anion radical quenching activity of W-LI extract from *Lawsonia inermis *leaves. Regression analysis: **represents significance at p≤ 0.01 One way ANOVA: F ratio = 47.72*; HSD = 12.89 (*p ≤ 0.05).

**Figure 5:**
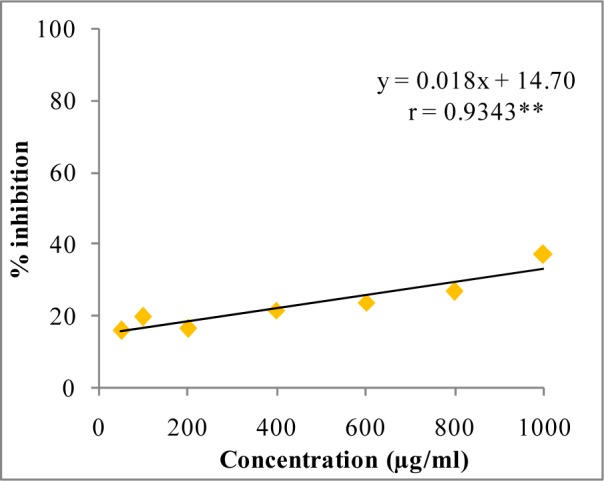
Hydroxyl radical quenching activity of W-LI extract from *Lawsonia inermis *leaves. Regression analysis: **represents significance at p≤ 0.01 One way ANOVA: F ratio = 80.88*; HSD = 0.06 (*p ≤ 0.05).

**Figure 6:**
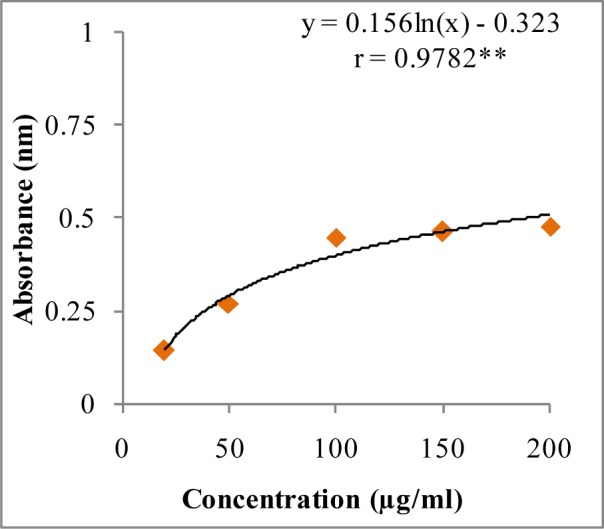
Reducing potential of W-LI extract from *Lawsonia inermis *leaves. Regression analysis: **represents significance at p≤ 0.01 One way ANOVA: F ratio = 103.44*; HSD = 3.88 (*p ≤ 0.05).

**Figure 7:**
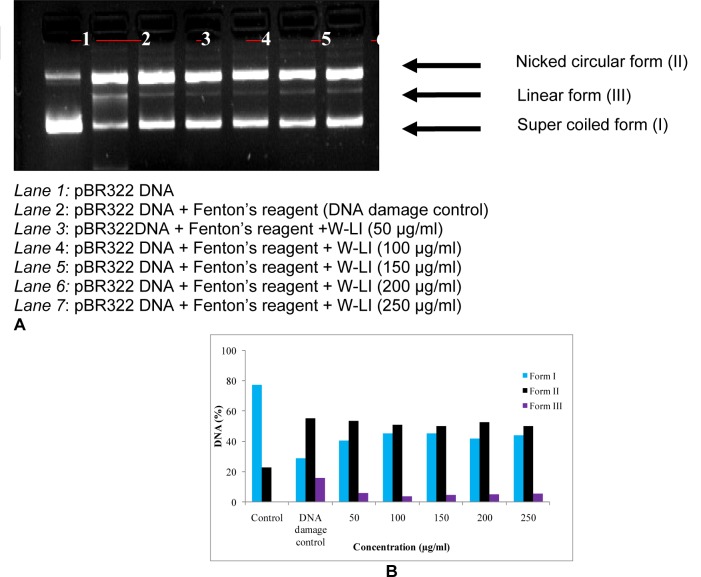
Figure 7A and B: Effect of W-LI extract from *Lawsonia inermis *on the protection of supercoiled pBR322 DNA against hydroxyl radical generated by Fenton's reagent.

In DPPH assay, W-LI showed moderate DPPH radical scavenging activity with 8.63 % at minimum tested concentration and 72.80 % at maximum concentration (Figure 2(Fig. 2)). In ABTS cation radical scavenging assay, W-LI exhibited weak effects at lowest tested concentration whereas at highest concentration it achieved inhibition percentage of 85.57 (Figure 3(Fig. 3)). Further, we found that W-LI extract moderately inhibited superoxide anion radicals. It showed radical scavenging ability of 48.44 % at of 1000 µg/ml (Figure 4(Fig. 4)). In specific deoxyribose degradation assay, W-LI demonstrated good hydroxyl radical scavenging activity of 59.75 % at 1000 µg/ml (Figure 5(Fig. 5)). Results were found to be statistically significant in both assays. In FRAP assay, W-LI exhibited an absorbance of 0.14 nm at minimum concentration (20 µg/ml) and 0.47 nm at highest tested concentration of 200 µg/ml (Figure 6(Fig. 6)). Further results of pBR322 plasmid DNA protection assay demonstrated DNA protective effects of W-LI as it showed 44.20 % native supercoiled form at highest concentration (250 µg/ml) while DNA damage control showed 29 % supercoiled form (Figure 7(Fig. 7)). Results reflected that W-LI has DNA scission inhibitory activity as it prevented the scission of plasmid DNA from hydroxyl radicals.

### Cytotoxic activity

**Figure 8:**
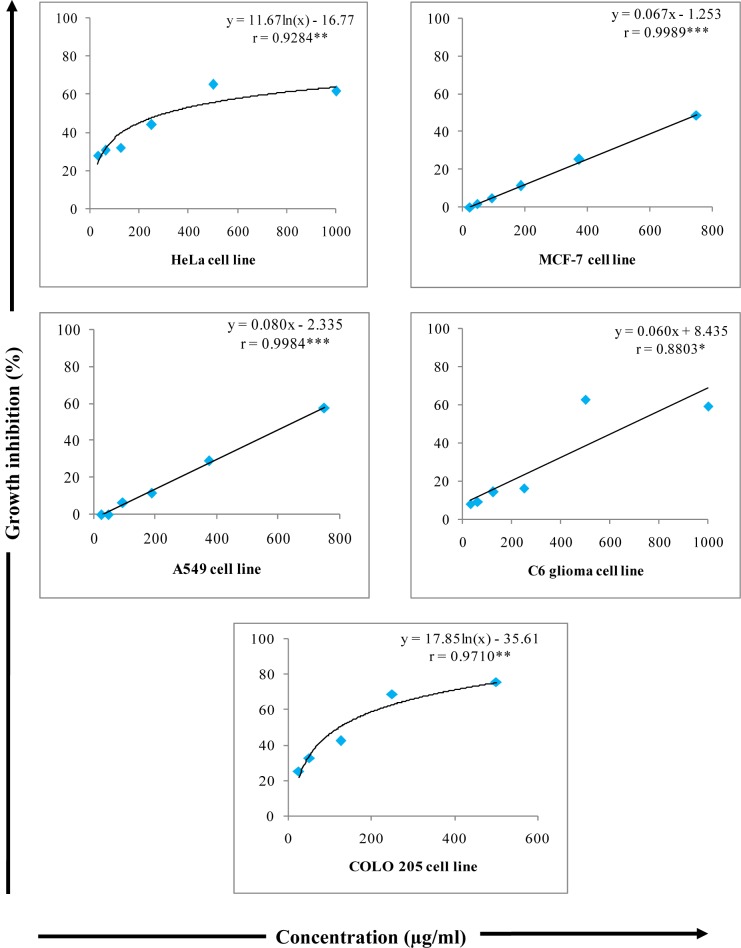
Growth inhibitory effects of various concentrations of W-LI from *Lawsonia inermis *leaves on cancer cell lines in MTT Assay. Regression analysis: *represents significance at p≤ 0.05; **represents significance at p ≤ 0.01; ***represents significance at p ≤ 0.001 One way ANOVA: For HeLa: F ratio = 63.740*; HSD = 9.69; For MCF-7: F ratio = 95.31*; HSD = 9.10); For A549: F ratio = 78.55*; HSD = 12.02; For C6: F ratio = 127.19*; HSD = 10.76; For COLO 205: F ratio = 86.41*; HSD = 11.06 (*p ≤ 0.05).

In present investigation, W-LI showed moderate antiproliferative effects of 61.68 % at 1000 µg/ml against HeLa cells. Extract inhibited 28.06 % of cell growth at minimum dose (31.25 µg/ml) (Figure 8(Fig. 8)). In MCF-7 cells, no antiproliferative effects were observed at lowest concentration whereas at highest concentration 48.63 % of cytotoxicity was observed (Figure 8(Fig. 8)). Extract showed moderate growth inhibitory effect against A549 cells with percent cytotoxicity of 57.73 (Figure 8(Fig. 8)). In C6 glioma cell line, W-LI inhibited growth of cells by 59.43 % (Figure 8(Fig. 8)). In COLO 205, activity was 25.19 % at lowest concentration (25 µg/ml) and 75.49 % at highest concentration (500 µg/ml) (Figure 8(Fig. 8)).

## Discussion

In the last few decades, numerous phytoconstituents with several health promoting properties have been explored (Khoudja et al., 2014[46]). Discovery of number of anticancer agents from the plants like vincristine, taxol, camptopthecin etc. have highlighted the use of natural plant products in cancer chemoprevention (Raskin et al., 2002[69]). Understanding the fact that medicinal plants are boon to mankind and are never ending repository of drugs, number of plant extracts are being tested for their various bioactive properties such as radical scavenging and anticancer activities (Widowati et al., 2013[83]). Further, drugs obtained from plants are known to be economical, easily available and are safe in nature (Fennell et al., 2004[26]; Farias, 2004[25]; Meena et al., 2009[62]; Ashraf et al., 2015[8]), which make them popular among people. Among different types of plant secondary metabolites, polyphenols are largest and widespread group of phytoconstituents throughout the plant kingdom. Phenolics are meant for defense of plant against photosynthetic stress, oxidative stress and eating by herbivores. Polyphenols are also consumed by human population in the form of fruits and vegetables. Among various polyphenolic constituents in the diet, most abundant are phenolic acids and flavonoids (Link et al., 2010[57]). These are found in various parts of plant *viz*. stems, roots, leaves, bark, flowers, fruits, seeds etc. (Iacopini et al., 2008[39]; Benayad et al., 2014[10]; de Andrade et al., 2014[19]). In the current investigation, W-LI was found to contain moderate amount of phenolic and flavonoids content. Further analysis using HPLC revealed the presence of different polyphenols as gallic acid, catechin, chlorogenic acid, caffeic acid epicatechin, umbelliferone, coumaric acid, rutin, ellagic acid, quercetin and kaempferol in W-LI. Ellagic acid was found to be major polyphenol in the extract. 

ABTS^.+^ and DPPH radicals are most commonly used chromogens for the determination of antioxidant activity of biological materials (Arnao, 2000[6]). W-LI was found to be promising inhibitor of both DPPH and ABTS cation radicals with an IC_50 _of 352.77 and 380.87 µg/ml respectively. Numerous studies in the literature have highlighted the antioxidant activity of above identified polyphenolic phytochemicals. Zhang et al. (2006[88]) reported antioxidant activity of various phenolic compounds viz. quercetin, ferulic acid, sinapic acid, rutin, catechin, caffeic acid and chlorogenic acid in ORAC and lipid peroxidation inhibition capacity assays. Cheel et al. (2007[14]) tested DPPH radical scavenging activity of the extracts obtained from *in vitro *cultures of *Sanicula graveolens* and reported that root culture showing highest chlorogenic acid content exhibited highest radical scavenging activity. Gallic acid and its derivatives were evaluated for DPPH radical scavenging activity by Kasture et al. (2009[43]) and reported that gallic acid showed highest radical scavenging potency among its various derivatives. Quercetin, gallic acid and methyl dehydrochebulate from *Phyllanthus niruri* were tested for DPPH radical scavenging activity by Ahmeda et al. (2009[2]) and reported that all three compounds demonstrated excellent antioxidant activity.

Superoxide anion radicals are one of the most powerful reactive oxygen species known to be involved in various pathophysiological activities and possess ability to give rise to other radicals by various reactions (Halliwell and Gutteridge, 1984[35]; Al-Mamun et al., 2007[3]). Superoxide radicals can give rise to hydroxyl radicals which can cause lipid peroxidation or can directly instigate lipid peroxidation (Wickens, 2001[82]). Other ROS which can be generated by superoxide anion radicals include hydrogen peroxide, hydroxyl radical and singlet oxygen. These different types of ROS and oxidizing entities can cause damage to various biomolecules such as protein, lipids and DNA (Pietta, 2000[66]; Klaunig et al., 2010[48]). W-LI was found to be moderate radical scavenger of superoxide anions and hydroxyl radicals. Results were found to be dose-dependent in both assays. The superoxide anion and hydroxyl radical scavenging activity of W-LI may be in part attributed to the polyphenols (ellagic acid, catechin, quercetin, kaempferol etc.) present in it. Zheng et al. (2009[89]) isolated different polyphenolic compounds such as ellagic acid, gallic acid, corilagin, ethylgallate, 1-β-*O*-galloyl-d-glucopyranose, methyl brevifolin carboxylate and 4-*O*-α-l-rhamnopyranosyl-ellagic acid from the seeds of Longan (*Dimocarpus longan* Lour) and reported that all the compounds with excellent superoxide anion radical scavenging capacity with IC_50_ ranging from 1.04-7.03 µg/ml. *Terminalia paniculata *and *Madhuca longifolia *extracts were evaluated for their comparative antioxidant effects (Agrawal et al., 2011[1]). It was found that in DPPH, ABTS and hydrogen peroxide radical scavenging assays, *M. longifolia* demonstrated better antioxidant potential where as in nitric oxide and hydroxyl radical scavenging assays, *T. paniculata *displayed better effects. Extracts from both plants were found to be rich in phenolic, flavonoids, proanthocyanidins and gallotannins.

Direct correlation has been observed between antiradical activities and reducing capacity of some plant extracts (Tanaka et al., 1988[79]). Reducing activities are generally related with the presence of reductones which acts as antioxidant by breaching free radical chains by donation of hydrogen atoms (Duh et al., 1999[22]; Gordon, 1990[32]). Results of the FRAP assay demonstrated that W-LI extract possessed immense reducing potential as there is exhibited increase in the absorbance values along with increase in concentration. Results were obtained in the dose-dependent fashion. HPLC analysis revealed the presence of phenolics compounds in varying amounts which could be the possible explanation for reducing potential of W-LI extract. Singh et al. (2009[75]) studied leaf, fruit and seed extract of *Moringa oleifera* for antioxidant activity and reported that leaf extract possessed good reducing potential in reducing power assay. HPLC analysis of the extract demonstrated the presence of phenolics constituents such as gallic acid, chlorogenic acid, kaempferol, quercetin, ellagic acid, ferulic acid and vanillin. Soobrattee et al. (2005[77]) studied various polphenolic phytochemicals for reducing potential in FRAP assay. Gallic acid, ellagic acid, chlorogenic acid, quercetin, kaempferol, (-)-epicatechin and (+)-catechin exhibited FRAP value of 5.25, 4.39, 3.22, 7.39, 1.95, 2.90 and 2.47 mmol Fe (II)/L respectively. In pBR322 plasmid DNA protection assay, W-LI inhibited the DNA scission induced by the hydroxyl radicals generated from Fenton's reagent and provided protection to pBR322 plasmid DNA. Plant extracts rich in phenols possess potential to protect plasmid DNA from damaging effects of hydroxyl radicals (Lee et al., 2002[55]). da Silva et al. (2011[17]) studied DNA protective effects of *Anadenanthera colubrina*,* Libidibia ferrea *and *Pityrocarpa moniliformis* fruits. Results demonstrated that *L. ferrea* showed the highest DNA protective capacity of 82.41 ± 0.93 %, followed by *A. colubrina* (73.44 ± 14.30 %) and *P. moniliformis *(70.01 ± 8.60 %) at 500 µg/ml and reported that the high phenol content in these extracts was responsible for protection against DNA damage. Verma et al. (2009[80]) reported that ethyl acetate/polyphenolic fraction of *Moringa oleifera* (MOEF) protected pUC18 plasmid DNA against damaging effect of hydroxyl radicals. Fraction treatment (10 µg/ml) protected supercoiled form which was comparable to that of catalase (5U) and quercetin (50 µM). MOEF demonstrated the presence of gallic acid, chlorogenic acid, kaempferol, ellagic acid, rutin, ferulic acid and quercetin.

The constituents possessing potential to block or suppress the cancer cell proliferation *via* induction of apoptotic cell death are regarded as potent anticancer agents (Frankfurt and Krishan, 2003[28]). It is widely recognized that intake of vegetable, fruits or plants rich in antioxidant constituents can be linked with cancer prevention (Virgili et al., 2001[81]; Johnson, 2001[41]; Argolo et al., 2004[5]). Dietary phytoconstituents are encouraged as medicinal agents due to their safe nature, less toxicity and general acceptance (Fennell et al., 2004[26]; Farias, 2004[25]; Meena et al., 2009[62]; Dai and Mumper, 2010[18]; Ashraf et al., 2015[8]). Protective properties of plants can be ascribed to the variety of constituents such as phenols, flavonoids, anthocyanins etc. (Sanchez-Moreno et al., 1998[71]; Zhang and Wang, 2002[87]). In present investigation, W-LI showed moderate antiproliferative effects against HeLa cells., MCF-7, A549 and C6 glioma cells. However, extract showed promising growth inhibitory activity against COLO 205 cells with GI_50_ of 121.03 g/ml. Losso et al. (2004[58]) reported that ellagic acid demonstrated selectively inhibited growth and induced apoptosis in various cancer cell lines *viz*. Caco-2, MCF-7, Hs578T and DU 145 while being non-toxic to normal human lung fibroblast cells. Choi and Ahn (2008[15]) reported kaempferol as potential inducer of apoptosis *via* G2/M cell cycle arrest in MDA-MB-453. Song et al. (2014[76]) reported that kaempferol effectively inhibited EGFR/p38 signaling activation, reduced cyclin B1 expression and increased p21 expression along with PARP cleavage and induced apoptosis in human renal carcinoma (786-O and 769-P) cells. Lee et al. (2015[56]) reported that quercetin induced apoptosis through mitochondrial pathways by ROS mediated ERK activation in HL-60 cells. Treated HL- 60 cells showed activation of caspase 3, 8 and 9, cleavage of PARP, depolarization of mitochondrial membrane and activation of ERK pathway. 

## Conclusions

From the results, it is concluded that water extract of *L. inermis* leaves harbors different polyphenolic phytoconstituents which possess immense antioxidant, DNA protective and anticancer potential. As the extract demonstrated promising antioxidant and DNA protective activity, it can be used as natural antioxidant in various food products after proper experimental studies. Further *in vivo* studies using murine models are required to validate its antioxidant and anticancer activities. 

## Acknowledgements

This work was supported by the Council of Scientific and Industrial Research (CSIR) [38(1265)/10/EMR-II], New Delhi (India) and the UPE program of University Grants Commission (UGC), New Delhi. 

## Conflict of interest

The authors declare that they have no conflict of interest.
